# Comparison of loop-mediated isothermal amplification (LAMP) and PCR for the diagnosis of infection with *Trypanosoma brucei* ssp. in equids in The Gambia

**DOI:** 10.1371/journal.pone.0237187

**Published:** 2020-08-24

**Authors:** Lauren Gummery, Saloum Jallow, Alexandra G. Raftery, Euan Bennet, Jean Rodgers, David G. M. Sutton

**Affiliations:** 1 Weipers Centre Equine Hospital, School of Veterinary Medicine, College of Medical, Veterinary and Life Sciences, University of Glasgow, Glasgow, United Kingdom; 2 Gambia Horse and Donkey Trust, Sambel Kunda, The Gambia; 3 Institute of Biodiversity, Animal Health and Comparative Medicine, College of Medical, Veterinary and Life Sciences, University of Glasgow, Glasgow, United Kingdom; Universita degli Studi di Parma, ITALY

## Abstract

**Introduction:**

Infection of equids with *Trypanosoma brucei* (*T*. *brucei*) ssp. is of socioeconomic importance across sub-Saharan Africa as the disease often progresses to cause fatal meningoencephalitis. Loop-mediated isothermal amplification (LAMP) has been developed as a cost-effective molecular diagnostic test and is potentially applicable for use in field-based laboratories.

**Part I:**

Threshold levels for *T*. *brucei* ssp. detection by LAMP were determined using whole equine blood specimens spiked with known concentrations of parasites. Results were compared to OIE antemortem gold standard of *T*. *brucei*-PCR (TBR-PCR).

**Results I:**

Threshold for detection of *T*. *brucei* ssp. on extracted DNA from whole blood was 1 parasite/ml blood for LAMP and TBR-PCR, and there was excellent agreement (14/15) between tests at high (1 x 10^3^/ml) concentrations of parasites. Detection threshold was 100 parasites/ml using LAMP on whole blood (LWB). Threshold for LWB improved to 10 parasites/ml with detergent included. Performance was excellent for LAMP at high (1 x 10^3^/ml) concentrations of parasites (15/15, 100%) but was variable at lower concentrations. Agreement between tests was weak to moderate, with the highest for TBR-PCR and LAMP on DNA extracted from whole blood (Cohen’s kappa 0.95, 95% CI 0.64–1.00).

**Part II:**

A prospective cross-sectional study of working equids meeting clinical criteria for trypanosomiasis was undertaken in The Gambia. LAMP was evaluated against subsequent TBR-PCR.

**Results II:**

Whole blood samples from 321 equids in The Gambia were processed under field conditions. There was weak agreement between LWB and TBR-PCR (Cohen’s kappa 0.34, 95% CI 0.19–0.49) but excellent agreement when testing CSF (100% agreement on 6 samples).

**Conclusions:**

Findings support that LAMP is comparable to PCR when used on CSF samples in the field, an important tool for clinical decision making. Results suggest repeatability is low in animals with low parasitaemia. Negative samples should be interpreted in the context of clinical presentation.

## Introduction

Trypanosomiasis is a potentially fatal haemolymphatic disease causing acute profound anaemia or a chronic cachexic syndrome. The disease has a high prevalence in working equids across sub-Saharan Africa, where the extracellular parasites are commonly tsetse fly transmitted (*Glossina* spp.). Disease caused by parasites from the *Trypanozoon* subgenus (*Trypanosoma brucei* ssp.) can result in infection of the central nervous system, causing neurological abnormalities including ataxia, somnolence and inevitably death. In The Gambia trypanosomiasis is hyperendemic. It contributes to loss of productivity and a high reported mortality in equids, sufficient to exceed live birth rate, with the potential to affect significantly the welfare of reliant communities [1–3].

Disease phenotype varies depending on the species of parasite involved. Various *T*. *brucei* ssp., including *T*.*b*. *brucei*, *T*.*b*. *equiperdum* and *T*.*b*. *evansi*, as well as *T*. *vivax* and *T*. *congolense* have been identified in The Gambia [4,5]. The early stages of disease in equids are associated with non-specific signs of infection such as pyrexia, anaemia, diarrhoea and abortion. Occasionally dependent or genital oedema or dermal plaques may be seen. Without treatment animals can die acutely or disease can last from months to years, with weight loss leading to cachexia, or in the case of *T*. *brucei* ssp. progression to neurological disease [6–9].

Disease surveillance is limited and has historically been based on visualisation of parasites microscopically in blood smears, buffy coat examination or lymph node aspirate. Reported sensitivity is low (between 100–10000 parasites/ml, technique dependent) [10,11] and speciation based on morphology is unreliable [12]. *T*. *brucei* ssp. are indistinguishable by available molecular tests and therefore this manuscript refers to *T*.*b*. *evansi*, *T*.*b*. *equiperdum* and *T*.*b*. *brucei* collectively as ‘*T*.*brucei* ssp.’ from this point. Diagnosis of *T*. *brucei* ssp. is complicated further by periods of low circulating parasitaemia when the parasite is sequestered in the tissues [13,14], and is rarely achieved prior to onset of neurological signs. Consequently treatment is often delayed since this is dependent on recognition of non-specific clinical abnormalities.

In equids the current gold standard for ante-mortem diagnosis of trypanosomiasis is reported as PCR [15], with TBR-PCR having the greatest demonstrable sensitivity for *T*. *brucei* ssp. [16]. Molecular methods suggest that the prevalence of *T*. *brucei* ssp. in equids in The Gambia ranges from 14%-20% [4,5,9]. Due to the possibility of low levels of parasitaemia in infected individuals TBR-PCR on samples gathered in the field cannot be described as a definitive antemortem test [17].

Neurological disease has been confirmed by demonstration of parasites in the neuropil using immunohistochemistry in animals with clinical signs [18]. The disease is invariably fatal once parasites reach the central nervous system with available treatment reported to be ineffective [10].

Molecular techniques such as TBR-PCR are of limited use for field-based diagnosis in this susceptible population due to expense and the requirement for specialised equipment. A highly sensitive field-applicable molecular test would greatly improve quality and efficacy of disease surveillance, leading to earlier diagnosis, improved welfare and more informed treatment strategy.

Loop-mediated isothermal amplification (LAMP) for *T*. *brucei* ssp., originally developed by Notomi et al. [19], is resistant to biological contamination and therefore can be used on a variety of biological templates [20]. LAMP has been demonstrated to have a high sensitivity and specificity for *T*. *brucei* ssp. [20–23]. The technique relies on a *Bst* DNA polymerase with a high strand displacement activity under isothermal conditions resulting in rapid DNA amplification. Reagents can be dried, simplifying storage of kits in areas where electricity and cooling facilities are limited [24]. The test requires limited sample processing and provides a visual result, with no post-processing of the amplification product, rendering the test potentially applicable to use in a field-based laboratory.

LAMP for *T*. *brucei* ssp. (RIME-LAMP) targets the repetitive mobile insertion element (500 copies per haploid genome [25]), enabling detection at low concentrations of parasite, and has been evaluated in many studies [20,22,23,26–29]. LAMP has been suggested as part of a screening procedure in clinical cases of sleeping sickness in humans [30].

The aim of the current study was to evaluate the practicality and efficacy of LAMP as a field based diagnostic for equine trypanosomiasis. It was hypothesised that LAMP would be suitable for field use in clinical cases of equine trypanosomiasis in a resource poor region. The diagnostic performance of LAMP was hypothesised to be equivalent to TBR-PCR for the detection of *T*. *brucei* ssp. in equine blood.

## Materials and methods

### Threshold study

**Fig 1:**
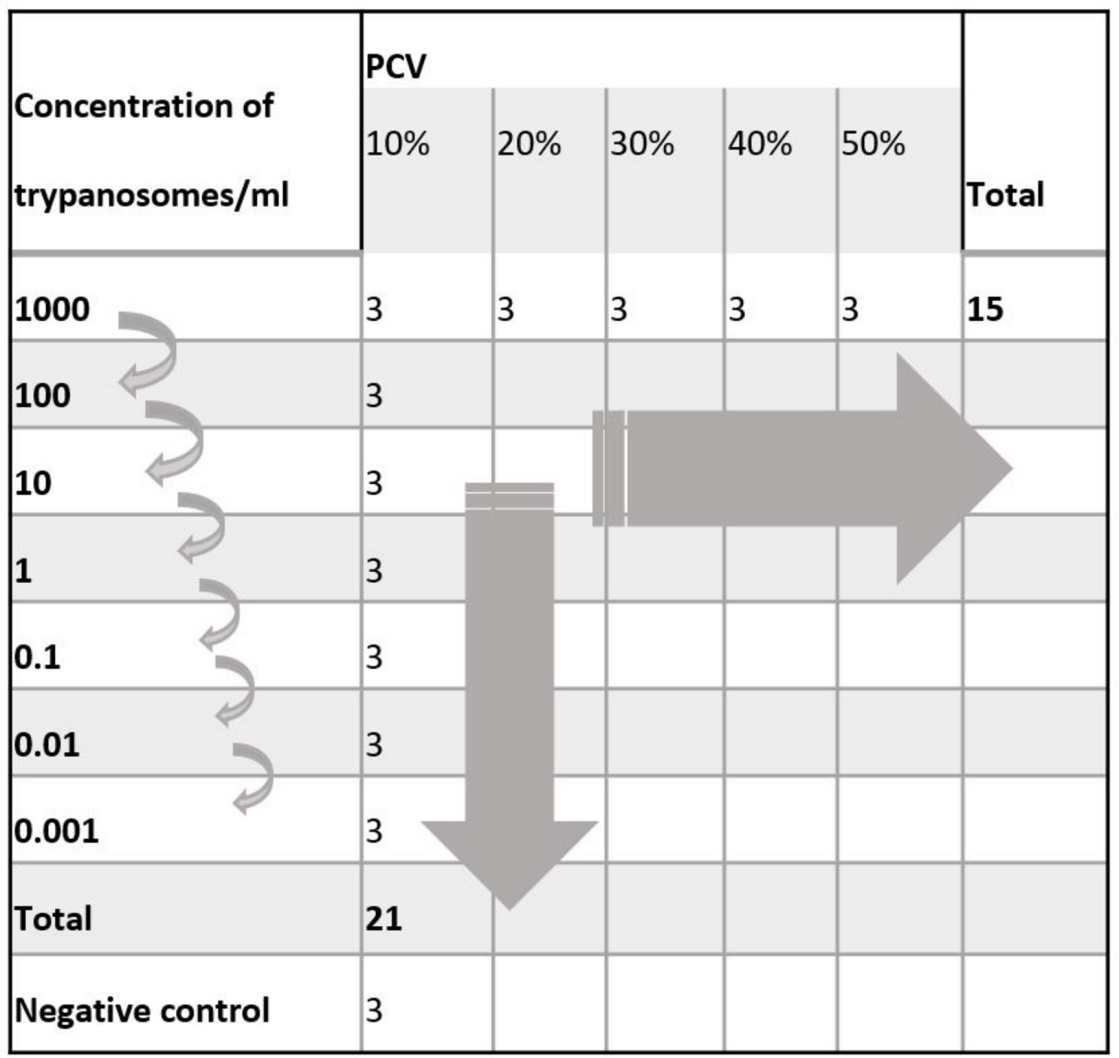
Sample table demonstrating design of *in vitro* experiment with whole blood containing serial dilutions of trypanosomes at a range of packed cell volumes.

A threshold *in vitro* validation study was employed to assess the analytical sensitivity of LAMP (RIME-LAMP) in diagnosing infection with *T*. *brucei* ssp. using whole equine blood template with known parasite concentrations. This was performed across a range of packed cell volumes to determine the effect of PCV on readability of the LAMP and was compared to TBR-PCR (Fig 1, Table 1).

**Table 1:** Molecular tests performed on each template.

Template	LAMP	TBR-PCR
Whole blood	✔	✖
Whole blood with detergent	✔	✖
DNA extracted from whole blood	✔	✔
DNA extracted from FTA cards	✔	✔

Samples were created at five packed cell volumes (PCV) from 10% to 50% by serial dilution (10x) each starting at 1 x 10^3^ parasites/ml until 1 x 10^−3^ parasites/ml. Each sample was then tested in triplicate to give a total of 21 samples at each PCV, and 15 at each parasite concentration.

### Serial dilution

Serial dilutions of *T*.*b*. *brucei* (strain 947 x 247 hybrid) were made using mouse blood containing 2 x 10^4^ parasites/ml. Whole defibrinated horse blood (E&O laboratories, Bonnybridge, Scotland) was prepared to provide packed cell volumes ranging from 10% to 50% at 10% increments by removing or adding a calculated amount of defibrinated serum. PCV values were confirmed using microhaematocrit centrifugation (HaemataStat II™, EKF Diagnostics, Cardiff, Wales) in duplicate.

Blood representing each adjusted PCV (1900 μl) was combined with 100 μl trypanosome infected mouse blood (containing 2 x 10^3^ trypanosomes) and serial dilutions were performed to make final concentrations between 1 x 10^3^ and 1 x 10^−3^ parasites/ml (Fig 1). A negative control was created by adding 60 μl mouse blood to 1140 μl equine blood at each PCV to mirror the constituents in the trypanosome spiked samples. Each sample was stored in EDTA, heparin and on FTA cards (100 μl). Spiked samples were processed at reducing trypanosome concentrations, and then tested in triplicate until the concentration at which all three samples were negative. Samples beyond this point in the dilution were not analysed. The negative control samples matching each PCV were also processed in triplicate.

### DNA extraction from whole blood

DNA was extracted from 200 μl of EDTA blood using the Genesig Magnetic Bead extraction kit (Primerdesign Ltd., Camberley, UK) according to manufacturer’s suggested protocol [31]. Final elution volume for the DNA was 200 μl.

### DNA extraction from FTA cards

DNA extraction from Whatman® FTA cards (GE Healthcare Ltd., Buckinghamshire, UK) was performed using QIAamp DNA MicroKit (Qiagen, Hilden, Germany) for gDNA extraction according to manufacturers’ instructions [32]. Extractions using 3 x 2mm punches per extraction were repeated in triplicate from each card to increase sensitivity of result [33]. Final elution volume was 30 μl.

### Preparation of LAMP template

LAMP was performed employing samples prepared by four different methods for use in the threshold study: (i) DNA extracted from whole blood; (ii) DNA extracted from FTA cards; (iii) whole blood using the ‘boil and spin’ method described below; (iv) whole blood using the ‘boil and spin’ method with added detergent.

Where extracted DNA template was used methods are as described above.

#### Boil and spin method

When preparing whole blood template for LAMP analysis using the ‘boil and spin’ method, 100 μl of heparinised blood was added to 900 μl PCR grade water and heated at 90°C for 10 minutes before centrifugation at 14000 rpm for 3 minutes. 200 μl of the resulting supernatant were stored at <5°C prior to further testing. LAMP was performed within 7 days of extraction.

When detergent was used 5 μl of 10% sodium dodecyl sulfate (SDS) solution was added to 45 μl of blood template. This was added to 450 μl PCR grade water before processing as described above.

### LAMP assay

Kits for LAMP were donated by Foundation for Innovative New Diagnostics (FIND, Geneva, Switzerland), produced for use in humans (Loopamp, Eiken Chemical Co. Ltd., Japan). The standard operating procedures for detecting *T*. *brucei* ssp. [34] were followed, with a total reaction volume of 25 μl, comprising 5 μl sample template (either extracted DNA or template from the ‘boil and spin’ method) and 20 μl of PCR grade water. A negative control (25 μl of PCR water) and positive control (25 μl of provided template) was included with each test run (<14 tubes). A water bath (Fisherbrand^TM^ Isotemp Waterbath, 2L, Fisher Scientific, Loughborough, U.K; laboratory) or LAMP incubator (LF-160 incubator, Eiken Chemical co, Taito-ku, Tokyo, Japan; field, Gambia) was used for incubation. The reaction was set for 40 min at 62°C. After completion, tubes were examined by an observer (LG) and then either inserted into the fluorescence unit (field; incorporated within the LAMP incubator) or examined with a handheld UV lamp (laboratory).

Specificity of LAMP has been demonstrated previously [20] and was not re-validated in this study.

### *T*. *brucei* ssp. PCR

PCR primers (Table 2) targeting a highly conserved region of equine cytochrome B (mitochondrial DNA) were used on all samples to confirm the presence of amplifiable DNA [35]. PCR primers (Table 2) targeting a multicopy, species specific region (177bp satellite region of the minichromosome) found in *T*. *brucei* ssp. were used (TBR1 and TBR2; [16]). Sequences are provided in Table 2. Anticipated specificity of primers was confirmed using BLAST [36] and these primers have been widely used for detection of *T*. *brucei* ssp.

**Table 2:** Primers used in PCR.

Primers	Sequence	Size of product
TBR1	GAATATTAAACAATGCGCAG	177bp [16]
TBR2	CCATTTATTAGCTTTGTTGC	
EqCyB Fw	GACCTACCAGCCCCCTCAAACATT	439bp [35]
EqCyB Rv	CTCAGATTCACTCGACGAGGGTAGTA	

PCR amplification was conducted in a total reaction volume of 25 μl containing 2.5 μl template DNA, 1 x HP Buffer containing 1.5 mM MgCl_2_ (ThermoFisher Scientific, Massachusetts, USA), 0.2 mM dNTPs (ThermoFisher Scientific, Massachusetts, USA), 0.5 μM forward primer, 0.5 μM reverse primer (Eurofins Scientific, Luxembourg) and 1.25 U Thermo-Start Taq DNA polymerase (ThermoFisher Scientific, Massachusetts, USA). Positive and negative controls were also included. Amplification was initiated by a single cycle of 15 min at 92°C. For equine cytochrome B primers this was followed by 35 cycles of denaturation for 15 s at 94°C, annealing for 30 s at 60°C and extensions for 90 s at 72°C. For *T*. *brucei* primers (TBR1 and TBR2) PCR assays were as above with the only alteration being a lower annealing temperature of 55°C. In all cases a final extension was included for 10 mins at 72°C.

All PCR from extraction of whole blood included Thermo-Start Taq DNA polymerase (ThermoFisher Scientific, Massachusetts, USA). DNA extracted from FTA cards was run using Qiagen HotStarTaq® Plus DNA Polymerase (Qiagen, Hilden, Germany). Reaction composition was the same and PCR protocols were changed based on manufacturer’s recommendations to an initial denaturation time of 5 min at 94°C. Amplification products were visualised on a UV transilluminator following electrophoresis through a 2% agarose gel containing 1 μg/μl ethidium bromide.

### Field study

#### Acquisition of samples in The Gambia

Field work was completed at 3 time points between November 2017 and December 2018 in locations across the Central River District in The Gambia. Owners were invited to present equids for examination and treatment. A history was obtained with the assistance of translators provided by Gambia Horse and Donkey Trust. Verbal informed consent was obtained from the owner prior to inclusion of their animal in the study.

On presentation each animal was examined by an experienced equine vet for signs consistent with trypanosomiasis. Body condition was scored [37], age was estimated from dentition and a jugular blood sample was taken for measurement of packed cell volume (PCV %) and total plasma protein (TP g/l) in order to assess hydration, degree of anaemia and requirement for treatment. Centrifugation of micro-haematocrit capillary tubes was used to measure PCV (HaemataStat II™, EKF Diagnostics, Cardiff, Wales). TP was measured on a handheld refractometer (Optika®, Ponteranica, Italy) that had been calibrated using deionised water.

Excess blood was placed into EDTA and heparinised tubes as well as a Whatman FTA^®^ Classic card. Samples were discarded following measurement of PCV and TP if the animals did not meet trypanosomiasis inclusion criteria (detailed below). Blood was stored at <5°C prior to analysis.

Animals were included in the study if at least two of the following inclusion criteria were fulfilled: body condition score ≤1.5/5; PCV ≤25%; temperature horse >38.5°C, temperature donkey >37.8°C; limb, genital or ventral oedema; a history of abortion at any time prior to the study, or neurological disease. Additional animals were included if a single criterion provided strong clinical suspicion of trypanosomiasis. Animals that were reported as having treatment with trypanocidal medication within a 4-week period prior to presentation or those with any debilitating condition likely to result in death or euthanasia during the study period were excluded from the study. However, these animals received veterinary care as indicated by their presenting signs. If euthanasia was indicated it was discussed and performed with the permission of the owner using methods locally available to preserve the welfare of the animal.

Included animals were identified with a microchip (standard placement) and were treated with isometamidium via a slow i.v. jugular injection (Intromidium, Interchemie, Holland, 0.5 mg/kg of 0.5% solution). Drugs were sourced from a verified reputable supplier. CSF samples were obtained aseptically from the lumbosacral region of animals presenting with characteristic signs of neurological trypanosomiasis using standard technique to enable clinical staging [38]. Included animals also received treatment for any concurrent condition, including administration of non-steroidal anti-inflammatories, anthelmintic, or antimicrobials as indicated. Animals were examined at 2 weeks to record response and any side effects of treatment and then were re-examined at 3, 9 or 12 months after initial sampling. If additional samples were taken paired testing was performed and included in analysis.

#### Processing of field-acquired samples

*In vivo* assessment of *T*. *brucei* ssp. infection status was performed by LAMP analyses at the field-base in The Gambia. TBR-PCR could not be performed on site therefore results were subsequently compared to TBR-PCR completed in the laboratory following DNA extraction in the field, or from FTA card specimens extracted in the laboratory.

At the field site DNA extraction and preparation of LAMP template was carried out as described above except the centrifugation step which was limited to 6000 rpm due to equipment available in the field. CSF, processed using the ‘boil and spin’ method, was additionally tested from field-acquired samples when available.

Whole blood and LAMP templates were stored at <5°C prior to processing; extracted DNA was transported to University of Glasgow, Scotland and stored at -20°C prior to analysis.

Repeat LAMP assays were performed at the field site on field acquired processed whole blood samples to determine whether additional test positive animals would be identified. These were randomly distributed across the individuals sampled.

### Ethics statement

Animal use (infected mouse blood) was authorized in the United Kingdom under the Animals (Scientific Procedures) Act 1986 and approved by the University of Glasgow Ethical Review Committee.

Ethical approval for the field study was provided by University of Glasgow School of Veterinary Medicine Research Ethics Committee (Reference 39a/17), and the Gambian Ministry of Agriculture. Procedures were performed by trained veterinarians or local veterinary technicians and were of direct benefit to the animals (categorised under Veterinary Surgeons Act 1966). This was the criterion used by the committee for ethical approval and so the field study did not come under the Animals (Scientific Procedures) Act 1986.

### Statistical analysis

Number of tests required for adequate power was estimated using a simple nomogram [39] and reported sensitivity and specificity for LAMP from field based samples (93% sensitivity and 96.4% specificity [28]), and was estimated at 350–600 tests.

Statistical analysis was performed using SPSS (v.25 IBM Corporation, Armonk, NY, USA). For laboratory validation Cohen’s kappa analysis was used to assess agreement between results obtained using PCR and LAMP assays on different templates; results are reported with 95% confidence intervals. Kappa coefficients and levels of agreement follow stringent recommendations [40]. For samples obtained in the field descriptive statistics were calculated to describe the populations. Median and inter-quartile range were reported for continuous variables (confirmed as non-parametric using Shapiro-Wilk tests). Cohen’s kappa analysis was used as above to analyse agreement from the first result of each test only (disregarding technical test replicates). All tests performed on samples obtained over 1 month apart in individual animals were included in kappa analysis to increase power. Number of test positives is reported from the first result of each test only as a proportion of total samples tested.

Sensitivity is reported for each test at varying parasite concentrations during the threshold study, however due to the lack of a definitive ante mortem test for *T*. *brucei* ssp. infection in equids, sensitivity, specificity, negative predictive value (NPV) and positive predictive value (PPV) are not reported for field results.

## Results

### Threshold of detection for *T*. *brucei* ssp. by TBR-PCR and LAMP

**Fig 2:**
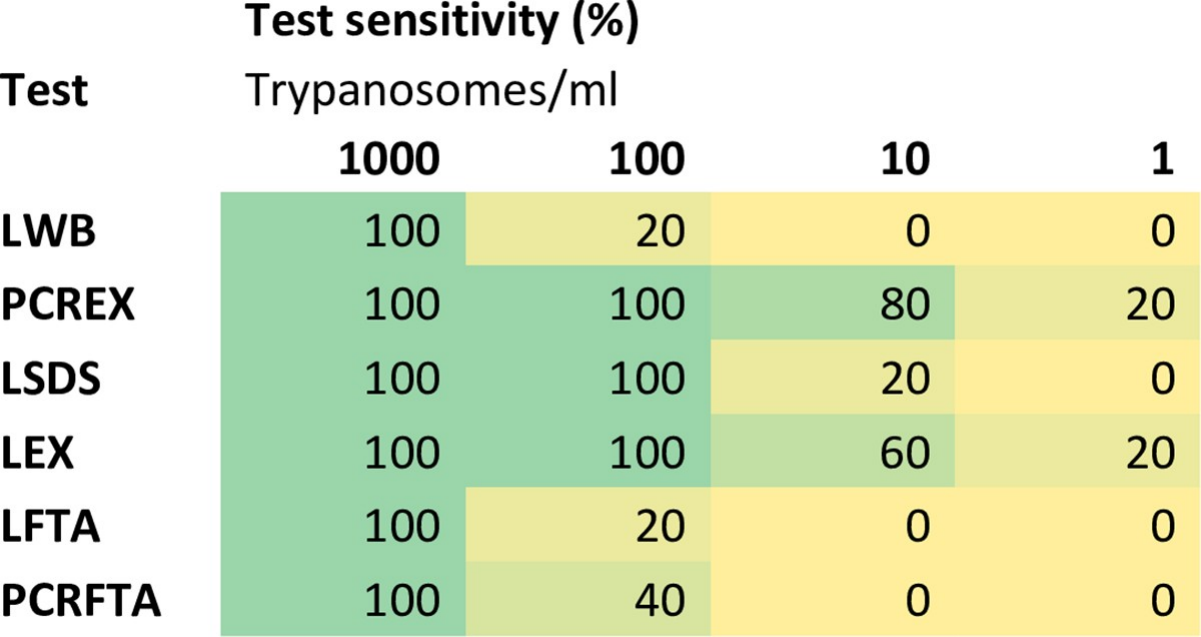
Threshold study: Test sensitivity at varying parasite concentrations in blood. The ability of various tests to detect trypanosomes at concentrations varying from 1000 trypanosomes/ml to 1 trypanosome/ml was assessed. The results are depicted in the form of a heat map with detection ranging from 100% to 0% across the various samples and tests. LWB: LAMP on whole blood template; LSDS: LAMP on whole blood treated with SDS detergent; LEX: LAMP on DNA extracted from whole blood; LFTA: LAMP on DNA extracted from FTA cards; PCREX: TBR-PCR on DNA extracted from whole blood; PCRFTA: TBR-PCR on DNA extracted from FTA cards.

The results obtained from the analyses performed on the four different sample categories: (i) LAMP on DNA extracted from whole blood (LEX); (ii) LAMP on DNA extracted from FTA cards (LFTA); (iii) LAMP on whole blood (LWB) and (iv) LAMP on whole blood treated with SDS detergent (LSDS) were compared with TBR-PCR on DNA extracted from whole blood (PCREX) and TBR-PCR on DNA extracted from FTA cards (PCRFTA). The relative thresholds for detection are represented in Table 3 and are reported as the concentration of parasites per millilitre in the primary blood sample prior to processing. The extrapolated concentrations of parasite in test samples are indicated in supporting information (S3 Table). There were no positive results from any test on negative control samples. At high parasite concentrations (1000 parasites/ml) all tests performed well, with LWB, LSDS and LEX detecting all positive samples (including all packed cell volumes, 5 samples at each concentration were processed in triplicate to give a total of 15/15 positive samples); PCREX detected 14/15, and this was reflected in a high sensitivity for all tests (Fig 2), and a good agreement between tests. LEX and PCREX had the highest analytical sensitivity, both detecting positives down to 1 parasite/ml. PCRFTA and LFTA had the lowest analytical sensitivity alongside LWB (100 parasites/ml). At lower concentrations parasite detection rate decreased, resulting in a lower sensitivity for all tests (Fig 2), and no test method identified trypanosome DNA at or below 0.1 parasites/ml. At 100 parasites/ml LEX detected more positive samples (14/15) when compared to PCREX (8/15), and LSDS detected more positive samples (15/15) than LWB (2/15).

**Table 3:** Laboratory threshold study: Number of positive results for each diagnostic test at a range of parasite concentrations.

Test	Sample	Primer set	Number of positive tests (total = 15)							
			Trypanosomes/ml							
			1000	100	10	1	0.1	0.01	0.001	-ve
LAMP	Whole blood	RIME	15	2	0	0	n.p.	n.p.	n.p.	0
	Whole blood SDS	RIME	15	15	3	0	n.p.	n.p.	n.p.	n.p.
	Extracted whole blood	RIME	15	14	5	1	0	n.p.	n.p.	0
	Extracted FTA card	RIME	11	1	0	0	0	n.p.	n.p.	n.p.
PCR	Extracted whole blood	TBR	14	8	5	1	0	n.p.	n.p.	0
	Extracted FTA card	TBR	12	2	0	0	0	n.p.	n.p.	n.p.

Three test replicates combining data at each PCV ranging from 10–50%, total = 15, n.p.; not performed.

#### Effect of PCV on test result

**Fig 3:**
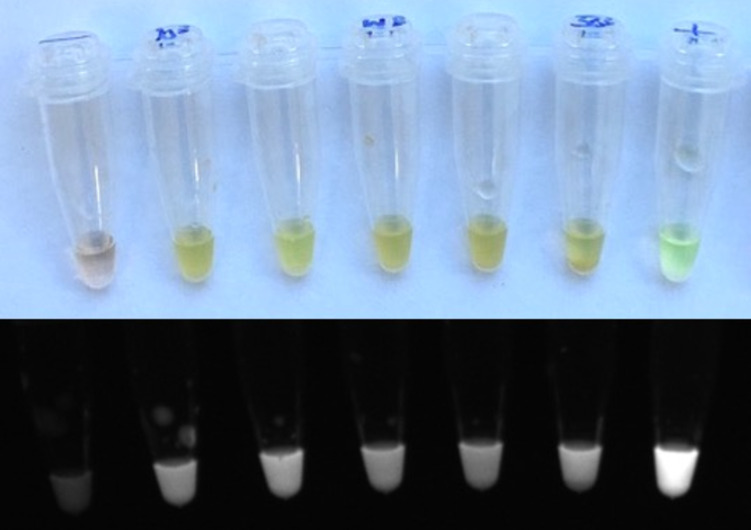
LAMP test result at range of PCVs at a concentration of 1000 parasites/ml in processed blood template. From left to right; negative control, 10% (+), 20%(+), 30% (+), 40% (+), 50% (+), positive control. Fluorescent result is visible in all test tubes. A small amount of cellular debris is visible at the bottom of the 50% tube (upper panel). This is confirmed by imaging in a UV transilluminator, where the positive results show fluorescence (lower panel).

**Fig 4:**
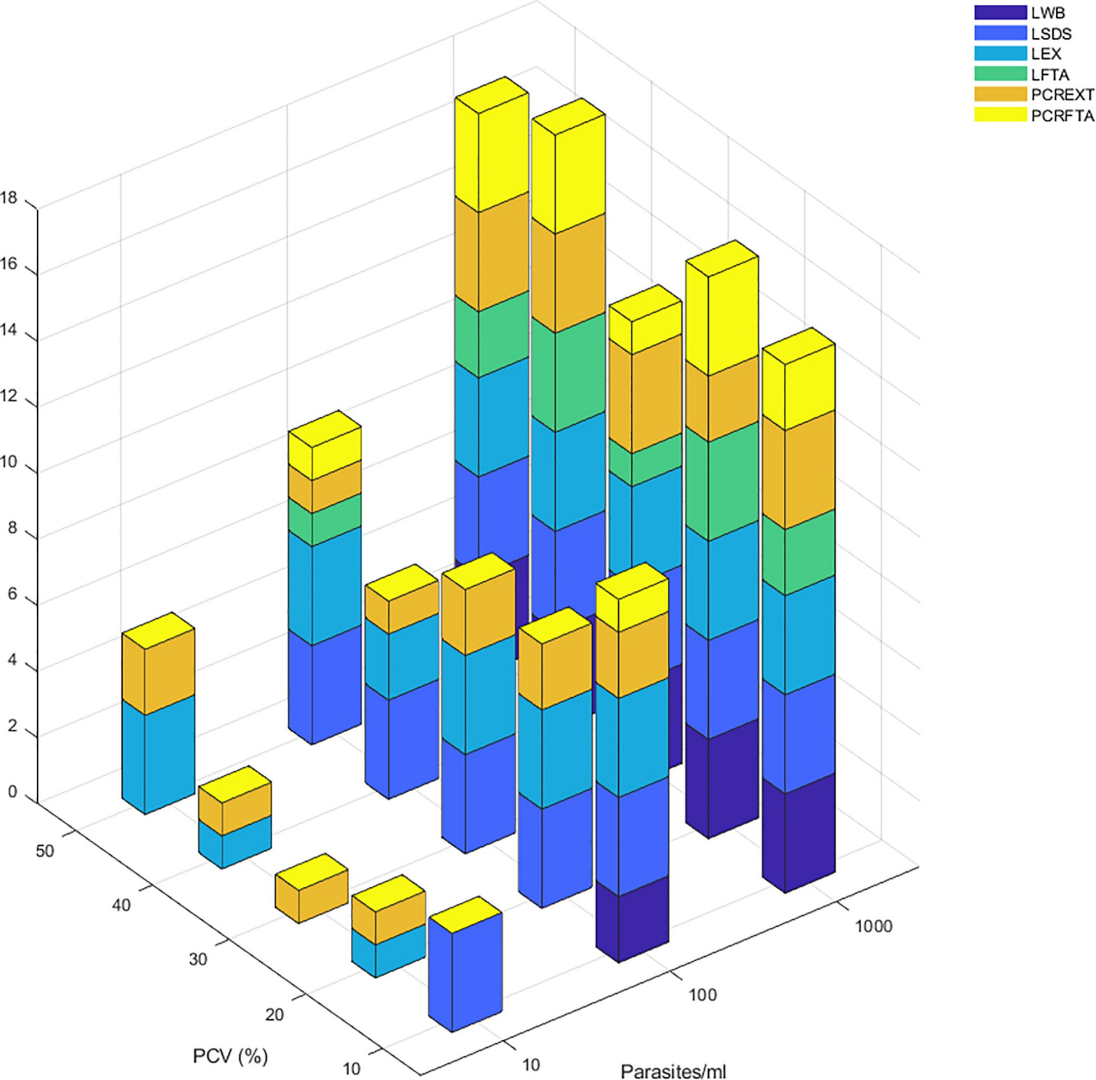
Threshold study: Number of test positives at a range of packed cell volumes at between 10 and 1000 parasites/ml in processed blood template. The number of positive results across the range of packed cell volumes (PCV; 10% to 50% at increments of 10%) is depicted by the height of the bars. Colour of the stacked bar represents type of test. Variability at the different packed cell volumes is present but generally inconsistent, with higher numbers of positive results at 10% and 50%. LWB: LAMP on whole blood template; LSDS: LAMP on whole blood treated with SDS detergent; LEX: LAMP on DNA extracted from whole blood; LFTA: LAMP on DNA extracted from FTA cards; PCREX: TBR-PCR on DNA extracted from whole blood; PCRFTA: TBR-PCR on DNA extracted from FTA cards.

During laboratory validation a visual result was achieved at all PCVs and was most easily identified using UV light (Fig 3). When all concentrations of trypanosome were considered at each PCV, results were subjectively more variable for LAMP across all categories (Fig 4). Higher numbers of positive results were seen across the tests at 10% and 50%, however PCV did not consistently influence test outcome.

### Test repeats on LAMP assays

**Fig 5:**
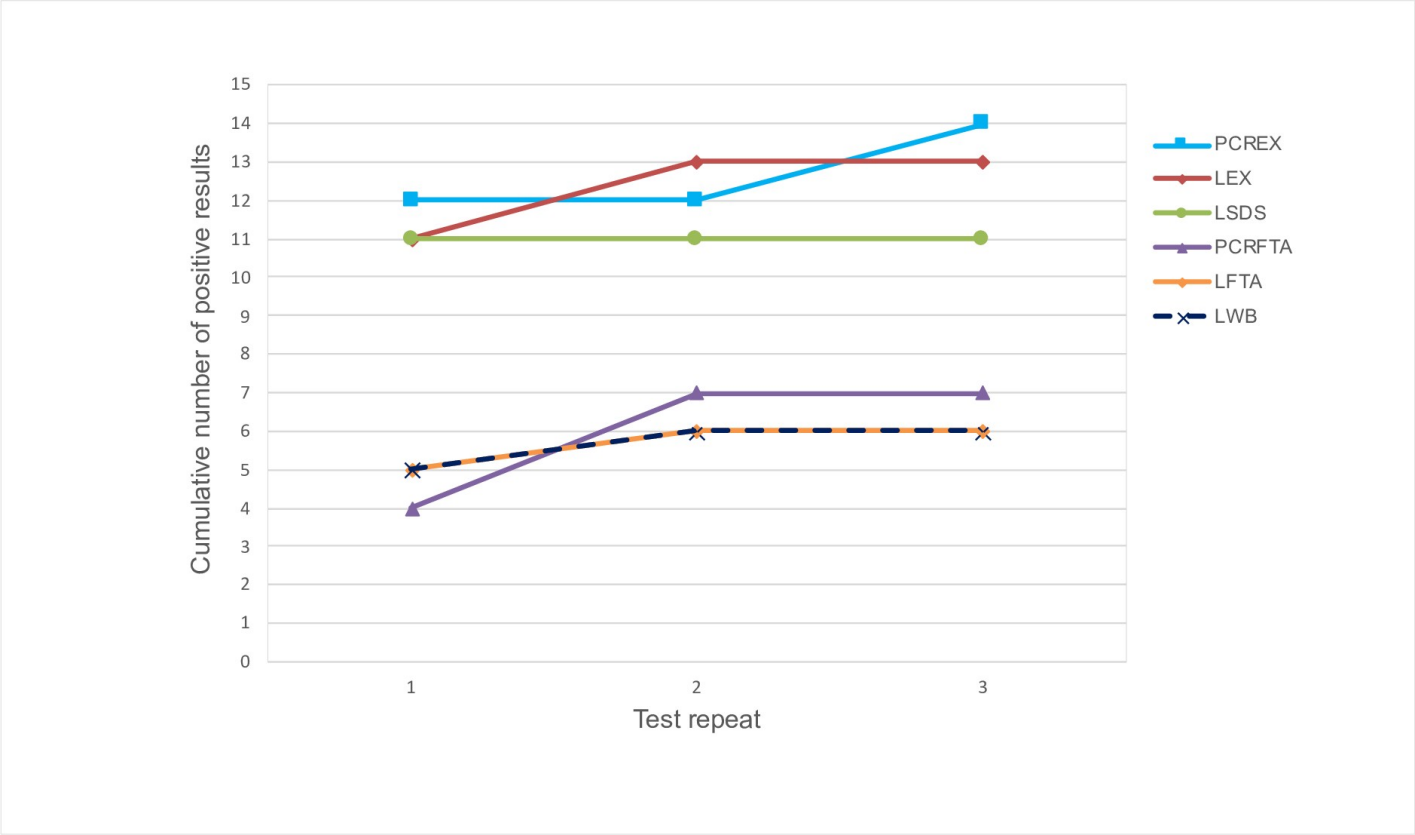
Threshold study: Chart showing cumulative positive test results over 3 rounds of analysis at concentrations above 10 parasites/ml (15 tests repeated in triplicate; n = 45). The number of test positive samples increases (between 1 and 3 additional positive samples) for all tests other than LSDS over 3 test replicates. Tests on DNA extracted from whole blood (PCREX and LEX) and LAMP on detergent treated blood (LSDS) appear to detect higher numbers of positive samples overall compared to those on DNA extracted from FTA cards (LFTA and PCRFTA) or LAMP on a whole blood template (LWB). LWB: LAMP on whole blood template; LSDS: LAMP on whole blood treated with SDS detergent; LEX: LAMP on DNA extracted from whole blood; LFTA: LAMP on DNA extracted from FTA cards; PCREX: TBR-PCR on DNA extracted from whole blood; PCRFTA: TBR-PCR on DNA extracted from FTA cards.

The cumulative positive result for each sample with a parasite concentration of over 10 parasites/ml was assessed over the three rounds of analysis (n = 15 to give a total of 45 tests). LEX, LWB, LFTA and PCRFTA detected 2, 1, 1, and 3 additional positives samples respectively on the second test round of analysis, but no further positive samples on the third test run (Fig 5). This resulted in an overall false negative rate of 9/15 for LWB and LFTA and for PCRFTA and LEX of 8/15 and 2/15 respectively. PCREX did not detect any additional positive samples on the second round of analysis however 2 more positive results were seen on the third test run resulting in a false negative rate of 1/15. LSDS was the only test with no additional positive samples detected in subsequent rounds of analysis, with a resultant false negative rate of 4/15.

### Cohen’s Kappa analysis

Cohen’s Kappa analysis (Table 4) across the range of serial dilutions showed almost perfect agreement between PCREX vs LEX (0.95, CI 0.64–1.00), but agreement was lower for PCREX vs LWB and PCREX vs LSDS. There was weak or minimal agreement between FTA card extracted DNA and whole blood extracted DNA using identical techniques (PCREX vs PCRFTA and LEX vs LFTA). LWB vs LEX and LWB vs LSDS had weak agreement whereas for LWB vs LFTA and LSDS vs LEX, agreement was moderate.

**Table 4:** Laboratory threshold study: Cohen’s Kappa result for each paired test and interpretation.

*Test 1 vs Test 2*	*Kappa value (95% CI)*	*Level of agreement*
PCREX vs LWB	0.45 (0.20–0.70)	Weak
PCREX vs LSDS	0.53 (0.16–0.90)	Weak
PCREX vs LEX	0.95 (0.64–1.00)	Almost perfect
PCREX vs PCRFTA	0.41 (0.10–0.72)	Weak
LWB vs LEX	0.53 (0.26–0.80)	Weak
LWB vs LSDS	0.57 (0.22–0.92)	Weak
LWB vs LFTA	0.78 (0.39–1.00)	Moderate
LEX vs LSDS	0.61 (0.24–0.98)	Moderate
LEX vs LFTA	0.34 (0.03–0.65)	Minimal

Levels of agreement; <0.20: None; 0.21–0.39: Minimal; 0.40–0.59: Weak; 0.60–0.79: Moderate; 0.80–0.90: Strong; >0.90: Almost perfect [40].

LWB: LAMP on whole blood template; LSDS: LAMP on whole blood treated with SDS detergent; LEX: LAMP on DNA extracted from whole blood; LFTA: LAMP on DNA extracted from FTA cards; PCREX: TBR-PCR on DNA extracted from whole blood; PCRFTA: TBR-PCR on DNA extracted from FTA cards.

### Field capability of LAMP

**Fig 6:**
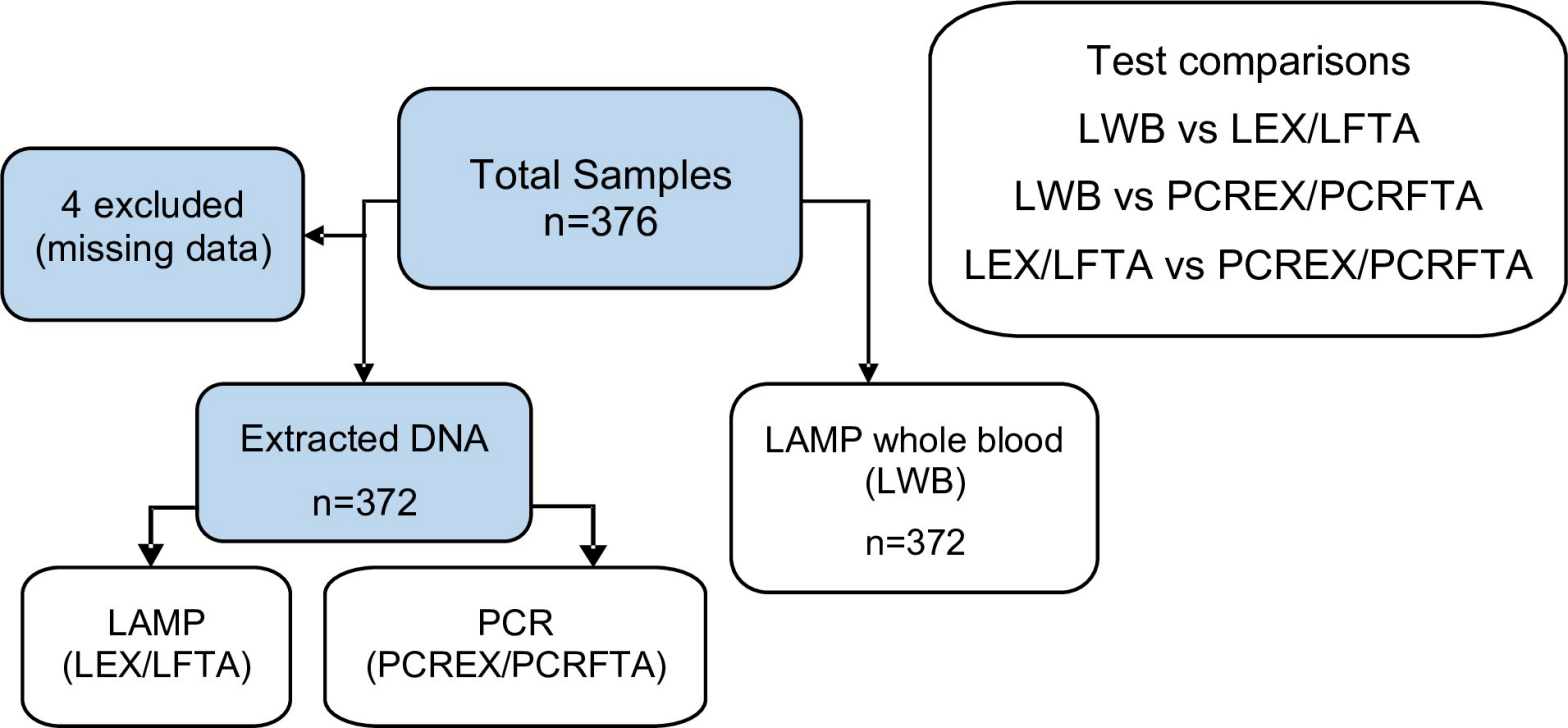
Field study: Diagram of samples analysed. Total number of included field-acquired samples was 372 after exclusions. LAMP analysis on whole blood (LWB), DNA extraction from whole blood and FTA card application were performed in the field. LEX, LFTA, PCREX and PCRFTA were performed in the laboratory. Comparisons between results (Cohen’s kappa) were performed between LWB and laboratory tests, and also between LAMP and PCR results from the same DNA samples. LWB: LAMP on whole blood template; LSDS: LAMP on whole blood treated with SDS detergent; LEX: LAMP on DNA extracted from whole blood; LFTA: LAMP on DNA extracted from FTA cards; PCREX: TBR-PCR on DNA extracted from whole blood; PCRFTA: TBR-PCR on DNA extracted from FTA cards.

Animals were examined from 13 villages across the central river district of The Gambia. Descriptive data are summarised in supplementary data (S1 Table). A total of 510 horses and donkeys were examined, and 315 animals fulfilled clinical inclusion criteria for trypanosomiasis: 114 horses (36.2%) and 201 donkeys (63.8%). 48.3% (n = 152) animals were female, 51.1% (n = 161) were entire male and 0.6% (n = 2) were gelded males. Median age was 5 years (range 3 months to 25 years), with a median body condition score of 2/5 (range 0.5-3/5) [37]. Median PCV was 25% (IQR 22–28%). Follow up data were included in order to increase power. Data were included from 41 animals at a follow up of either 3 months (29) and 12 months (20) or 9 months (12) to give a total of 376 paired tests. Four animals were subsequently excluded due to missing data to give a total sample size of 372 tests (Fig 6). For 183/372 samples, DNA was extracted from the corresponding FTA cards for analysis due to concern about low level contamination at the time of whole blood extraction. Following processing LAMP template (whole blood) for field analysis had subjectively more haemoglobin content due to lower centrifugation speeds.

#### Analysis of field-acquired samples

Prevalence using each individual test was similar to or lower than those previously reported (Table 5). LWB and PCREX resulted in similar numbers of positive tests (9.1% and 9.7% respectively), however there was a higher number of test positive animals when results were combined (17.7%) due to incomplete overlap. Combinations of positive results are detailed in supplementary data (S2 Table).

**Table 5:** Field study: Total number of positive *T*. *brucei* ssp. test results (n = 372).

*Test positives*	*All samples (% of test results positive)*
Any test positive	65/372 (17.5%)
LAMP WB (LWB)	34/372 (9.1%)
LAMP extracted DNA (LEX/LFTA)	33/372 (8.9%)
PCR extracted DNA (PCREX/PCRFTA)	36/372 (9.7%)

Results from DNA extracted from whole blood (first result, excluding test replicates) and those from substituted DNA extracted from FTA cards were combined to give total positive results for the tested population.

LWB: LAMP on whole blood template; LEX: LAMP on DNA extracted from whole blood; LFTA: LAMP on DNA extracted from FTA cards; PCREX: TBR-PCR on DNA extracted from whole blood; PCRFTA: TBR-PCR on DNA extracted from FTA cards.

Six CSF samples were initially obtained from 5 animals and showed 100% agreement (5/5 of initial samples positive on LAMP using whole CSF template and TBR-PCR on extracted DNA). One animal was resampled 3 months after treatment and was negative on both tests.

#### Cohen’s Kappa analysis on field-acquired samples

Cohen’s Kappa was used to compare test agreement (Table 6). Agreement was minimal between LWB vs LEX/LFTA (0.39, 95% CI 0.17–0.49) or LWB vs PCREX/PCRFTA (0.34, 95% CI 0.19–0.49). When analysing data from FTA cards in isolation, agreement between PCRFTA vs LWB (n = 179) was 0.23 (95% CI 0.03–0.43). When comparing PCREX vs LWB (n = 193) Cohen’s kappa was 0.46 (95% CI 0.25–0.69).

**Table 6:** Field study: Cohen’s Kappa result for each paired test detecting *T*. *brucei* ssp. in working equids and interpretation (n = 372).

*Test 1 vs Test 2*	*Kappa value (95% CI)*	*Level of agreement*
LWB vs PCRFTA/PCREX	0.34 (0.19–0.49)	Minimal
LWB vs LFTA/LEX	0.39 (0.17–0.49)	Minimal
PCRFTA/PCREX vs LFTA/LEX	0.57 (0.42–0.72)	Weak

Levels of agreement; <0.20: None; 0.21–0.39: Minimal; 0.40–0.59: Weak; 0.60–0.79: Moderate; 0.80–0.90: Strong; >0.90: Almost perfect [40].

LWB: LAMP on whole blood template; LEX: LAMP on DNA extracted from whole blood: LFTA: LAMP on DNA extracted from FTA cards; PCREX: TBR-PCR on DNA extracted from whole blood; PCRFTA: TBR-PCR on DNA extracted from FTA cards.

#### Test repeats of LAMP assays on field-acquired samples

**Fig 7:**
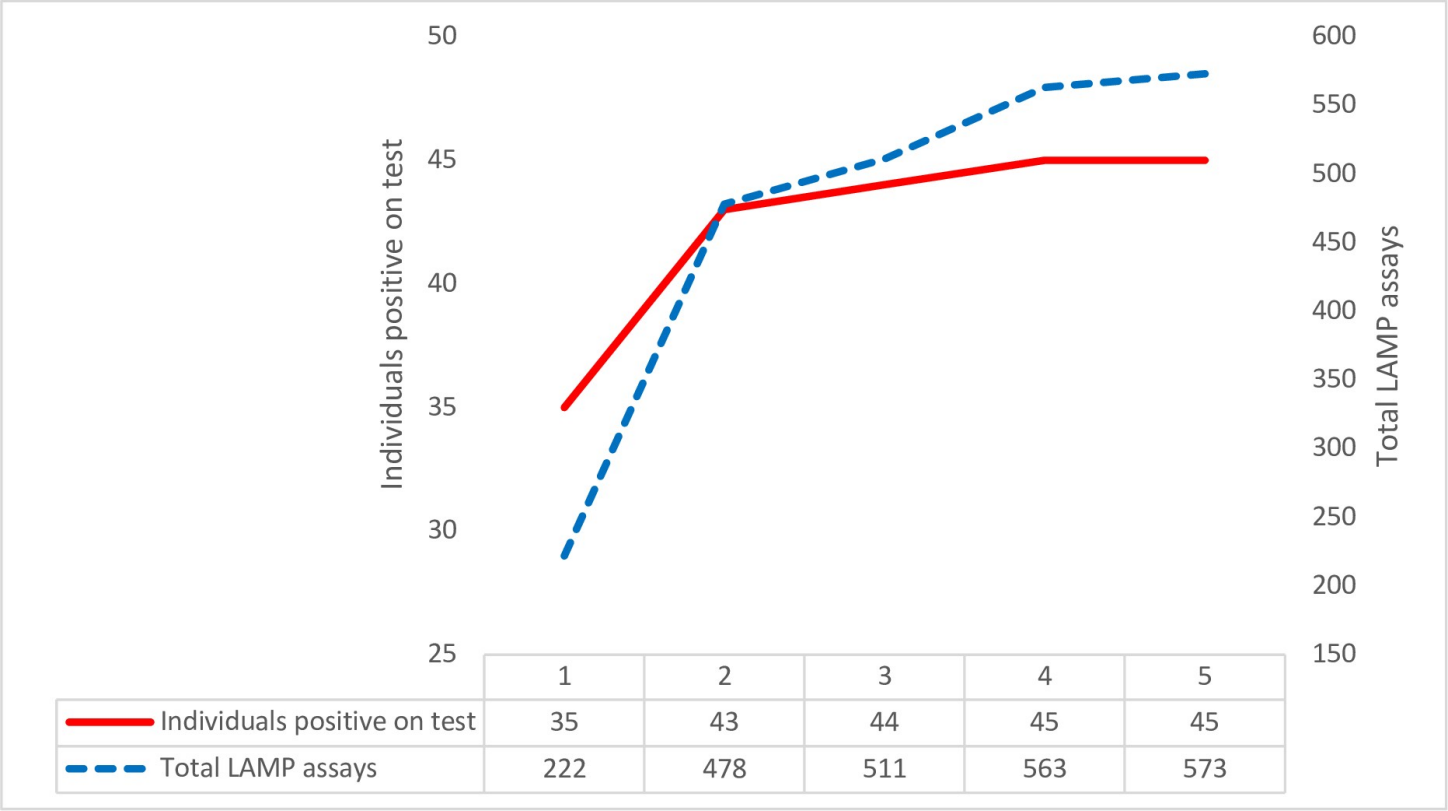
Field study: Chart to show cumulative positive results compared to number of repeat LAMP assays on a single sample. The number of individuals with a positive test results (red) is plotted against the total number of test replicates performed (blue, LAMP test on whole blood; LWB). The number of positive individuals increases up to 4 test replicates but starts to plateau between 2 and 4 test replicates.

Due to expected low parasitaemia, one or more test repeats were performed on 154/376 LWB assays on whole blood in the field to assess expected increase in detection of positive animals (Fig 7). Repeat tests were performed on the same template and were randomly distributed across the individual samples. 128 assays were repeated twice, 11 three times, 13 four times and 2 five times. 35 animals had a positive result on first LAMP assay. Prevalence increased up to a total positive result of 45 animals (45/315, equating to a prevalence of 14.3%). Number of additional positive individuals was observed to plateau with repeat tests.

## Discussion

This is the first study evaluating field-use of LAMP for the diagnosis of *T*. *brucei* infection in equids. The laboratory threshold study describes the analytical detection limit of LAMP using DNA extracted from whole equine blood by field-applicable methods. This was higher than previously reported for RIME-LAMP which has been described at 100 trypanosomes/ml with 4 primers [20] compared to 1 trypanosome/ml in this study (Table 3). DNA contained in 1 parasite is estimated at 0.1pg [41]. Therefore, although higher sensitivities of TBR-PCR have been reported previously (down to 0.1 trypanosomes/ml) [16] using these field-applicable methods the detection limit of LAMP equalled that of TBR-PCR. Quantification of DNA following sample processing was not possible as part of this study.

Performance of LAMP on DNA extracted from whole blood exceeded TBR-PCR at 100 parasites/ml with fewer false negative results, indicating LAMP is likely to detect animals with the same level of parasitaemia. Previous studies suggest the sensitivity of LAMP to be equal to or greater than PCR [22,42,43], although in this study the greater test volume (5μl compared to 2.5μl for TBR-PCR) is likely to have contributed to this result. The addition of 2 further primers has been reported to increase the sensitivity of LAMP further, with a positive result at only 0.001 trypanosomes/ml [20]. Test sensitivity decreased for both tests at or below 10 parasites/ml and when testing DNA extracted from FTA cards, despite processing FTA card samples in triplicate. Parasite DNA on FTA cards is likely to be fixed and unevenly distributed across the card, leading to a stochastic sampling effect and possible underestimation of prevalence [33].

Positive test results were observed using LAMP on a whole blood template (LWB) with a detection limit of 100 parasites/ml which was improved to 10 parasites/ml by the addition of SDS (LSDS) in the laboratory threshold study. Inclusion of detergents causing cell lysis has been described in whole blood template, CSF and prior to application of blood to FTA cards and improves sensitivity of LAMP [26,44]. This step was not included in the field study here due to the increased risk of sample contamination. However, LAMP on SDS treated samples has been used for *T*.*b*. *gambiense* detection in human CSF with promising results [44], and could be evaluated in field-based studies in equids in the future.

Detection of positive samples in the threshold study increased with test repeats, and this was most evident at low concentrations of parasites. In all samples treated with SDS the first LAMP test result was predictive of replicate results, potentially suggesting cell lysis reduces variability in test results due to a lower likelihood of sampling error [26]. Previous studies have used three test replicates to formulate a basis for results [26], and results in this study demonstrated 100% sensitivity following 3 repeats for both PCR and LAMP in samples containing above 100 parasites/ml when using DNA extracted from whole blood. Sensitivity of LAMP on whole blood template was also 100% at 1000 parasites/ml, but fell to only 20% at 100 parasites/ml, which was the limit of detection for this template. This could be associated with a lower total amount of parasite DNA within the sample, inhibition of the reaction by haemoglobin or a reduced ability to detect a positive result in templates prepared from whole blood as reported in previous studies [24,45], although the effect of PCV was not replicated in the comparison between blood samples with varying packed cell volumes (Fig 4).

Paired LAMP tests using different templates but with similar paired analytical sensitivities (LEX vs LSDS and LWB vs LFTA) had higher levels of agreement according to Cohen’s kappa. Low parasite concentration (which increases the chance of sampling error) and low prevalence conversely can affect Cohen’s Kappa negatively [26,44,46]. This should be considered when interpreting Cohen’s Kappa result for paired samples.

No negative controls displayed positive results during the threshold study. Specificity of primer binding in both RIME-LAMP and TBR-PCR is high as demonstrated previously [20,42]. Overall, sensitivity and specificity of TBR-PCR is reported generally as high (summary values of 99.0% and 97.7%), although specificity was variable (55.6%-82.9%) across multiple studies using PCR on satellite targets [17]. The low values reported for specificity in those studies is likely to have resulted from adoption of microscopic identification of parasites as the reference standard. In a study using pooled spiked tsetse fly midguts, specificity of RIME-LAMP was higher than TBR-PCR (75% and 26% respectively), but false positive LAMP results have also been reported occasionally [22].

When used under field laboratory conditions, LAMP was successful in detecting *T*. *brucei* ssp. infection in working equids and was particularly sensitive in confirmation of CNS trypanosomiasis in animals presenting with neurological signs. Test result interpretation of LAMP on whole blood template was subjectively more challenging in field conditions due to lower centrifugation speeds resulting in more haemoglobin transfer into the template, complicating direct comparison with the supplied positive control. This may have decreased sensitivity for recognition of a positive result [45]. Studies have described different methods for improving detection of test result such as the addition of hydroxynaphthol blue [47], and this approach could be trialled in future studies.

When results from both tests on field-acquired samples were combined, a higher number of individuals were classified as positive (17.5%) than for either individual test. One explanation for this is that each test is selecting different positive cases based on alternative target sequences for amplification; TBR-PCR targets a satellite sequence with 1000 copies [48] while RIME-LAMP targets 500 copies/haploid genome [25]. Alternatively, either test may be generating false positive or negative results.

Reduced prevalence of disease in field acquired samples was observed using TBR-PCR test results alone compared to previous studies in this population [4,9]. One study used whole genome amplification prior to PCR [4], the other used larger volume whole blood extraction [9] possibly increasing sensitivity. If prevalence is underestimated in this study it may be explained by methods, template type or preparation resulting in different DNA yields or the presence of inhibitors. Processes were limited by those applicable to field conditions, minimising cost, waste and contamination risk. Animals with clinical signs indicative of trypanosome infection, that returned a negative result on analysis, may have been infected with other species of trypanosome (*T*. *vivax* or *T*. *congolense*), and mixed infections have been reported [4,9] however testing for all species was beyond the scope of this study.

Test agreement between TBR-PCR and LAMP was excellent on CSF samples (6/6), potentially due to large quantities of parasite DNA in the CSF of neurological individuals, or reduction in inhibitors resulting in improved test sensitivity with this template. LAMP could therefore be recommended for disease staging in the field in animals with neurological signs of disease or positive for *T*. *brucei* ssp. on whole blood analysis. Accurate staging of this disease is considered vital in humans [49] and could be important in equids as treatment with non-CNS penetrant trypanocidal agents in CNS-stage infections have been shown to exacerbate the neuroinflammatory reaction associated with trypanosome infection in a murine model [50]. No medications have yet been shown to be successful at treating the neurological stage of disease in equids.

When testing field-acquired samples, agreement was weak for TBR-PCR and LAMP. The substitution of DNA extracted from FTA cards rather than from blood for the PCR may have resulted in a lower sensitivity and agreement (as demonstrated in the threshold study). Low levels of parasitaemia could also have influenced test agreement. In a previous field-based study test agreement between RIME-LAMP and 18S PCR on samples obtained in the field from humans with positive parasitological diagnosis was good (Cohen’s Kappa 0.61, 95% CI 0.45–0.77), but in suspected cases (high CATT or trypanolysis positive with no parasitological diagnosis, and presumably with lower level parasitaemia) was only minimal (Cohen’s Kappa 0.39, 95% CI 0.22–0.56) [28].

TBR-PCR is likely to result in false negative test results when parasitaemia in infected animals falls below the limits of detection [13]. Due to the lack of a definitive gold standard test for the diagnosis of *T*. *brucei* ssp. infection ante mortem, the specificity of LAMP could not be confirmed in this study. Definitive diagnosis may be achieved by post-mortem and immunohistochemistry [18], but was not possible in this population.

In this field study, as in the threshold study, up to 3 test repetitions resulted in the identification of additional positive individuals. In screening the general population, the possibility of a lower prevalence of disease and its effect on the interpretation of results should be considered [51]. A sensitive test is beneficial, but increased test repeats could potentially increase the risk of false positive results. The plateau reached in this study at higher numbers of repeat assays suggest that additional positives are unlikely to be a function of the number of test repeats. Future field studies could incorporate SDS to improve LAMP sensitivity over fewer test repeats [44].

## Conclusion

The findings of this study support the application of LAMP as a suitable screening test for use in the field, providing a means of *T*. *brucei* ssp. diagnosis in resource poor regions. *T*. *brucei* ssp. LAMP detection rates are comparable to TBR-PCR when used on samples with high parasitaemia, and on CSF samples under field conditions, offering an important additional tool for clinical decision making. The use of detergents is recommended for future field trials to help increase sensitivity and repeatability in LAMP, and negative samples should be interpreted with caution in the context of clinical presentation.

## Supporting information

S1 Table Field study: Clinical parameters of included population at the point of inclusion (n = 315). Data are provided for the population as a whole and divided into species. Values are given as median and interquartile range and signalment is comparable to that reported in previous studies in this population [9].
(DOCX)

S2 Table Table to show number of results for each combination of positive tests in field-acquired samples (total positive samples n = 65). Results are from tested samples collected and processed under field laboratory conditions from animals with suspected trypanosomiasis. Results from tests on DNA extracted in the field or from DNA extracted from FTA cards are combined.
(DOCX)

S3 Table Demonstration of effect of sample preparation for different testing modalities on potential trypanosome content of test substrate. Quantity of parasite is extrapolated by volume from number of parasites in initial blood sample. Parasite DNA is reported as 0.1pg DNA/ trypanosome [41].
(XLSX)
