# Supplementary material for: Comparison of loop-mediated isothermal amplification (LAMP) and PCR for the diagnosis of infection with Trypanosoma brucei ssp. in equids in The Gambia
Source: PLoS One. 2020 Aug 24;15(8):e0237187. doi: 10.1371/journal.pone.0237187 (PMC7444819; doi:10.1371/journal.pone.0237187)
Supplement: S1 Table — Data are provided for the population as a whole and divided into species. Values are given as median and interquartile range and signalment is comparable to that reported in previous studies in this population [9]. (DOCX) [file pone.0237187.s001.docx]

| **Parameter** | **Donkey (n=201)** | **Horse (n=114)** | **Combined (n=315)** |
| --- | --- | --- | --- |
| **Age (estimated; yrs)** | 5.0 (2.5-12.0) | 4.0 (1.0-15.0) | 5.0 (1.4-14.0) |
| **Body condition score (/5;[36])** | 2 (1.5-2.25) | 1.5 (1-2) | 2 (1.4-2.5) |
| **Gender**  **Male**  **Female**  **Neutered male** |  |  |  |
|  | 103/201 (51.24%) | 58/114 (50.88%) | 161/315 (51.11%) |
|  | 97/201 (48.26%) | 55/114 (48.25%) | 152/315 (48.25%) |
|  | 1/201 (0.01%) | 1/114 (0.01%) | 2/315 (0.01%) |
| **Weight (estimated; Kg)^1^** | 119.0 (103.0-130.0) | 200.0 (176.5-240.0) | 130.0 (110.0-185.0) |
| **Packed cell volume (%)^2^** | 25 (21-27) | 26 (22-29) | 25 (22-28) |
| **Total protein (g/l)** | 75 (69-80) | 70 (62-80) | 73 (67-80) |

^1^Estimated from validated nomograms [1, 2].

^2^Range used for packed cell volume (PCV) and total protein (TP) were donkey PCV 27-42%, TP 58-76g/l [3]; horse PCV 31-43%, TP 53-73g/l [4].

References

1. Carroll CL, Huntington PJ. Body condition scoring and weight estimation of horses. Equine Vet J. 1988;20: 41–5. Available: http://www.ncbi.nlm.nih.gov/pubmed/3366105

2. Pearson RA, Ouassat M. Estimation of the liveweight and body condition of working donkeys in Morocco. Vet Rec. 1996;138: 229–233. doi:10.1136/vr.138.10.229

3. Burden FA, Hazell-Smith E, Mulugeta G, Patrick V, Trawford R, Brooks Brownlie HW. Reference intervals for biochemical and haematological parameters in mature domestic donkeys (Equus asinus) in the UK. Equine Vet Educ. 2016;28: 134–139. doi:10.1111/eve.12512

4. Rossdales Laboratories. Reference Ranges, Adult Non-Thoroughbred Horses. 2016 [cited 16 May 2020]. Available: https://www.rossdales.com/assets/files/Adult-Non-Thoroughbred-Horses.pdf
