# Supplementary material for: Comparison of loop-mediated isothermal amplification (LAMP) and PCR for the diagnosis of infection with Trypanosoma brucei ssp. in equids in The Gambia
Source: PLoS One. 2020 Aug 24;15(8):e0237187. doi: 10.1371/journal.pone.0237187 (PMC7444819; doi:10.1371/journal.pone.0237187)
Supplement: S2 Table — Results are from tested samples collected and processed under field laboratory conditions from animals with suspected trypanosomiasis. Results from tests on DNA extracted in the field or from DNA extracted from FTA cards are combined. (DOCX) [file pone.0237187.s002.docx]

| **Test Result** | | | **Number in category** |
| --- | --- | --- | --- |
| **LWB** | **LEX/LFTA** | **PCREX/PCRFTA** |  |
| + | + | + | 12 |
| + | + | - | 3 |
| + | - | + | 2 |
| - | + | + | 9 |
| + | - | - | 17 |
| - | + | - | 9 |
| - | - | + | 13 |
| **Total** | | | **65** |

LWB: LAMP on whole blood template; LEX: LAMP on DNA extracted from whole blood; LFTA: LAMP on DNA extracted from FTA cards; PCREX: TBR-PCR on DNA extracted from whole blood; PCRFTA: TBR-PCR on DNA extracted from FTA cards. +: test positive, -: test negative.
